# Reasons and remedies for the agglomeration of multilayered graphene and carbon nanotubes in polymers

**DOI:** 10.3762/bjnano.7.109

**Published:** 2016-08-12

**Authors:** Rasheed Atif, Fawad Inam

**Affiliations:** 1 Northumbria University, Faculty of Engineering and Environment, Department of Mechanical and Construction Engineering, Newcastle upon Tyne NE1 8ST, United Kingdom

**Keywords:** agglomeration, carbon nanotubes (CNTs), dispersion state, multilayered graphene (MLG), polymer nanocomposites

## Abstract

One of the main issues in the production of polymer nanocomposites is the dispersion state of filler as multilayered graphene (MLG) and carbon nanotubes (CNTs) tend to agglomerate due to van der Waals forces. The agglomeration can be avoided by using organic solvents, selecting suitable dispersion and production methods, and functionalizing the fillers. Another proposed method is the use of hybrid fillers as synergistic effects can cause an improvement in the dispersion state of the fillers. In this review article, various aspects of each process that can help avoid filler agglomeration and improve dispersion state are discussed in detail. This review article would be helpful for both current and prospective researchers in the field of MLG- and CNT-based polymer nanocomposites to achieve maximum enhancement in mechanical, thermal, and electrical properties of produced polymer nanocomposites.

## Introduction

It has been well known for about hundred years that the addition of nano-fillers to polymers, polymer glasses, and semicrystalline polymers can remarkably improve the performance of polymers [1]. The important structural characteristics of nano-fillers include a large surface-to-volume ratio and a chemical texture of the surface [1–2]. Surfaces are inherently high-energy sites. Because nano-fillers have high a surface area, they also have very high values of surface energy. When the nano-fillers are added to a polymer matrix this high surface energy results in strong interfacial interactions. Polymer composite theory foretells improved mechanical properties due to improved interfacial bonding [3]. In addition, due to the high thermal and electrical conductivities of multilayered graphene (MLG) and carbon nanotubes (CNTs), thermally and electrically conductive polymer nanocomposites can be produced. However, all these enhancements of the performance of polymers can only be achieved when the filler is uniformly dispersed and no agglomeration of filler in the polymer matrix takes place.

MLG and CNTs have been reported to promote the proliferation of osteoblasts (bone-forming cells) and neurons, and found to be effective nano-carriers for several biomolecules such as proteins, DNA and carbohydrates [4]. Recently, MLG/CNT–polymer nanocomposites have been explored as scaffolds for cell growth and load-bearing implant materials for replacing defective human bones. However, some researchers reported that CNTs exhibit cytotoxicity to human dermal cells. Potential health hazards could also arise from inhalation. The discrepancy in such biocompatibility results can be attributed to the complicated physicochemical interactions between CNTs and biological cells, as well as to different methods to measure cell viability and different CNT sources. More efforts are needed to solve these issues prior to the incorporation of MLG/CNT–polymer nanocomposites into the human body. Therefore, it is a prerequisite to master the production of MLG- and CNT-based polymer nanocomposites and to gain knowledge about their biocompatibility and performance in living organisms.

One of the main issues in the production of polymer nanocomposites is the dispersion state of fillers, because MLG and CNTs tend to agglomerate due to van der Waals forces. The agglomeration can be avoided by using organic solvents, selecting suitable dispersion and production methods, and by functionalizing the fillers. Another proposed method is the use of hybrid fillers as synergistic effects can cause an improvement in the dispersion state of the fillers. In this review article, various aspects of each process that can help avoid filler agglomeration and improve the dispersion state are discussed in detail. This review article might be helpful for both current and prospective researchers in the field of MLG- and CNT-based polymer nanocomposites to achieve maximum enhancement in mechanical, thermal, and electrical properties of produced polymer nanocomposites.

## Review

### Graphene

Graphene, a two-dimensional densely packed honey-comb crystal lattice made of carbon atoms, has revolutionized the scientific parlance due to its exceptional physical, electrical, and chemical properties. Graphene, which is now found in various applications, was previously considered only a research material and a theoretical model to describe the properties of other carbonaceous materials such as fullerenes, graphite, single-walled carbon nanotubes (SWNT) and multiwalled carbon nanotubes (MWNT). It was believed that stand-alone single-layered graphene (SLG) could not exist in reality because of thermal fluctuations as the stability of the long-range crystalline order found in graphene was considered impossible at finite (room) temperatures. This notion was further corroborated by experiments that showed that the stability of a film decreases with decreasing film thickness [5]. However, graphene can be currently found on silicon substrates or suspended in a liquid and ready for processing. Although there are not many industrial applications of graphene, it is widely used for research purposes, e.g., as reinforcement in polymer matrix composites (PMC) and has shown to yield significant improvements in different (mechanical, thermal, and electrical) properties of the produced nanocomposites [6–9].

Graphene exhibits a honeycomb lattice, the sp^2^ bonding of which is much stronger than the sp^3^-bonding found in diamond [10]. A σ-bond is formed between the sp^2^-hybridized p*_x_* and p*_y_* orbitals [5] and the p*_z_* orbitals form π-bonds with half-filled bands that allow free motion of electrons. When graphene is bombarded with pure carbon atoms, hydrocarbons, or other carbon-containing molecules, the carbon atoms are inserted into vacancies thereby self-repairing holes in the graphene sheet. Faber and Evans, using crack deflection modeling, showed that, among all other nano-reinforcements, maximum enhancement in fracture toughness can be attained by employing graphene as reinforcement. This improvement in fracture toughness can be attributed to the higher capability of graphene to rebound advancing cracks [11–12].

**Figure 1:**
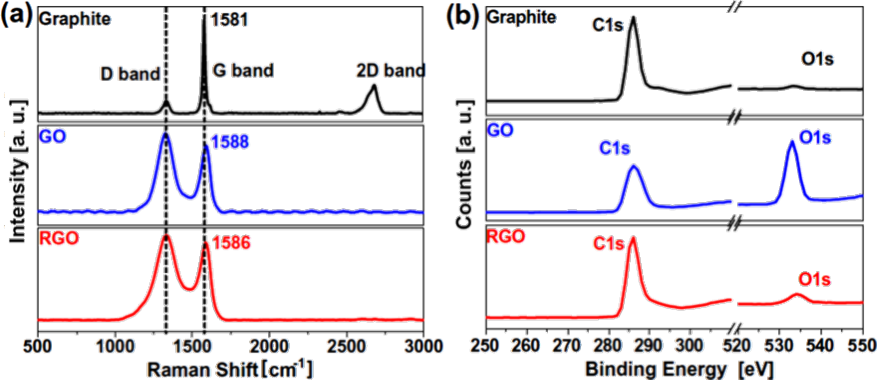
(a) Raman spectra and (b) XPS survey scans of graphite, graphene oxide and thermally reduced graphene oxide. Reproduced with permission from [13], copyright 2013 Elsevier.

**Figure 2:**
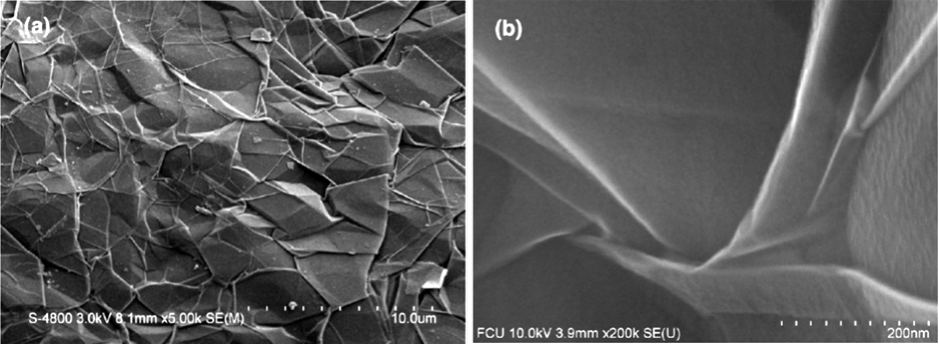
Wrinkling of multilayered graphene: (a) A typical wrinkling pattern. (b) A magnified view of the wrinkles. Reproduced from [16], copyright 2013 Elsevier.

The Raman and XPS spectra of graphite, graphene oxide (GO), and thermally reduced graphene oxide (RGO) are shown in Figure 1a,b. The graphene structure can be studied by using transmission electron microscopy (TEM) and other high-resolution imaging tools. Wrinkles were observed in flat graphene sheets that occur due to the instability of the 2D lattice structure [13]. Wrinkling is a large and out-of-plane deflection caused by compression (in-plane) or shear, and it is usually found in thin and flexible materials such as cloth fabric [14]. MLG was also found to undergo wrinkling [15]. When wrinkling takes place, strain energy is stored within MLG, which is not sufficient to allow MLG to regain its shape. Wrinkling can also be found in exfoliated graphite and a typical wrinkling pattern of exfoliated graphite is shown in Figure 2. The wrinkles in MLG are wider apart or closer together at different locations. As MLG does not store sufficient elastic strain energy, wrinkling is irreversible but it can be altered by external influences [16]. The surface roughness varies owing to the different topographical features such as size and shape of the wrinkles. Therefore, the ability of sheets to mechanically interlock with other sheets and polymer chains varies. Wang et al. showed that wavelength and amplitude of wrinkles are directly proportional to the volumetric dimensions of the graphene sheets as described in Equation 1 and Equation 2, where λ is the wrinkle wavelength, ν is Poisson’s ratio, *L* is the graphene sheet size, *t* is the thickness of the graphene sheet, ε is edge contraction on a suspended graphene sheet, and *A* is the wrinkle amplitude [17].

[1]
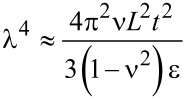


[2]
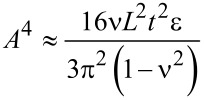


A great amount of energy is stored in the graphene sheets due to their coiled structure [18–19]. When an external load is applied, the graphene sheets undergo plastic deformation and a large amount of energy is absorbed [20]. Bending and folding takes place in graphene sheets and van der Waals interactions help to compensate the bending energy at the folds through intersheet adhesion [21–22]. Under thermal stress and external loading, the individual layers of graphene undergo crumpling [23–24], scrolling [25–26], folding [27–28], rippling [29–30], and out-of-plane wrapping [31–32], making graphene suitable to enhance the performance of polymers.

### Carbon nanotubes

CNTs were first discovered by Iijima in 1991, who produced multiwalled carbon nanotubes (MWNTs) through arc-discharge evaporation [33]. The synthesis of CNTs can be linked to the discovery of fullerene C_60_ (buckyball) in 1985 [34]. CNTs can be regarded as one dimensional carbon materials with aspect ratios greater than 1000 [35]. CNTs have density down to about 1.3 g·cm^−3^ [2]. The graphite planes in CNTs are rolled up in cylindrical shape with diameters at the nanoscale. The ends of CNTs are capped with hemifullerene [35]. Hemifullerene is more reactive than CNTs itself due to the increased curvature. It is analogous to polymeric end groups [36]. There are different opinions about the nature of CNTs. Different researchers have referred to CNTs as molecules, nanostructures, nanocolloids, particles, graphite cylinders and in one opinion, CNTs are just fibers [36]. The procedures applied for the production of CNTs also resulted in new structures with unique geometries and properties such as carbon nanohorns, cup-stacked CNTs (CSCNTs), carbon nanobuds or carbon nanotori [37]. The two most commonly used CNTs are single-walled carbon nanotubes (SWNTs) and MWNTs.

SWNTs, as indicated by the name, consist of a single layer of graphene forming a seamless cylinder. SWNTs were discovered in 1993 by Iijima and Ichihashi [38] and Bethune and co-workers [39]. The typical diameter range of SWNTs is from 0.4 to about 3 nm. SWNTs with a diameter smaller than 0.4 nm are thermodynamically unstable due to the strain induced by the curvature onto the carbon–carbon bonds. The attraction between the opposing ends of the graphene cylinder surmounts the radial stiffness as the cylinder diameter becomes larger (more than 3 nm) and cylinder tends to flatten [36]. Nanotubes, especially SWNTs, are held together in the form of ropes (aggregated nanotubes) [2]. The commercially available SWNT ropes usually have average diameters in the range of 10–20 nm and lengths of several micrometers [35]. These ropes have strengths of about 13–52 GPa (more than steel by a factor of 10) and tensile moduli of about 1 TPa (more than steel by factor of 5) [2].

**Figure 3:**
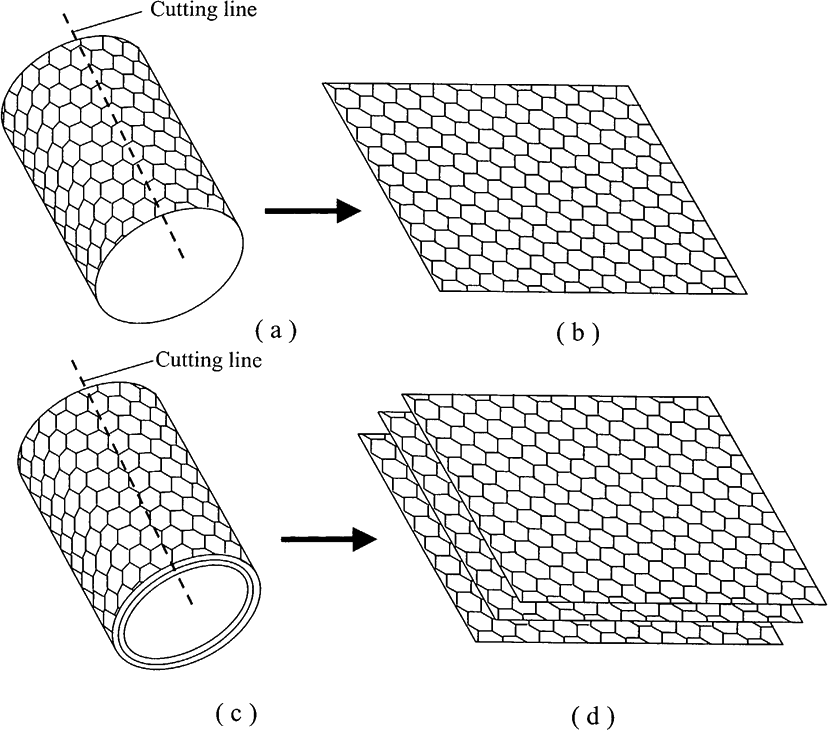
(a) SWNT, (b) cut and flattened SWNT consisting of single-layer graphene (SLG), (c) MWNT, and (d) cut and flattened MWNT consisting of multi-layer graphene (MLG). Source: itech.dickinson.edu.

MWNTs comprise a number of concentric graphene cylinders, which is known as “Russian doll” structure. There are van der Waals forces between adjacent graphene layers [40]. MWNTs have diameters and lengths in the ranges of 10–20 nm and of 10–50 μm, respectively [41]. It was observed that MWNTs fail at the outer tube with the interior showing a “sword and sheath” mechanism [2]. The schematics of SWNTs and MWNTs are shown in Figure 3.

### Conformation

**Figure 4:**
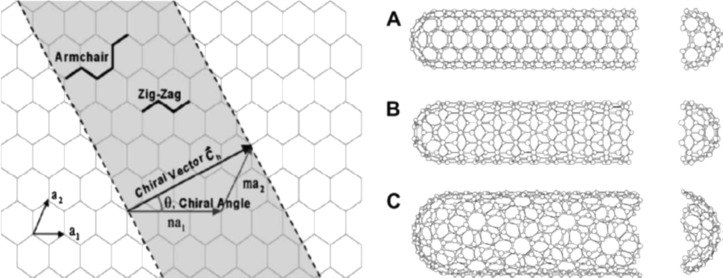
Schematics of graphene sheets rolled to form CNTs with different conformations (A: armchair, B: zigzag, C: chiral). Reproduced with permission from: left panel [42], copyright 2001 Elsevier; right panel [43], copyright 1995 Elsevier.

One important property of CNTs is their conformation. It depends on the angle at which the graphene sheet is rolled as shown in Figure 4 [36,40]. There are three main conformations with one conformation being helical, i.e., having axial chirality: (1) armchair, (2) zigzag, and (3) chiral. The geometry and/or chirality of a tube is defined by Equation 3, where *C*_h_ is the chiral vector, and *n* and *m* are the steps in the hexagonal lattice along the vectors *a*_1_ and *a*_2_, respectively [40].

[3]
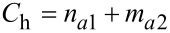


If *n* = *m*, the nanotube is called “armchair”. If *m* = 0, it is called “zigzag”, and in all other cases it is chiral. The chirality affects the transport properties, especially electronic properties. If (2*n* + *m*) is a multiple of 3, the nanotube exhibits electron transport properties of a metal, while it behaves as a semiconductor if above condition is not satisfied. The semiconducting CNTs have varying bandgaps [36]. The bandgap for semi-conducting CNT is inversely proportional to the diameter of nanotube. The values of about 1.8 eV and 0.18 eV have been reported of bandgap for small and large diameters nanotubes, respectively [2]. About two third SWNT behave as semiconductor while remaining as metals. It is interesting to note that in MWNT, each graphene layer can have different chirality [40]. Unfortunately, all the manufacturing methods of CNT produce nanotubes of varying geometries [36].

### Surface density

Sometimes it is preferable to mention the surface density of nanotubes. As CNTs are usually found in agglomerated form, it becomes nearly impossible to measure the surface density using electron microscopy. The volume density can be used to approximate the surface density by using Equation 4, where *m*_u_ is mass of a unit length of SWNTs with an average diameter of *d*_a_, *a*_C–C_ is the C–C bond length in a SWNT (1.44 Å), and *m*_c_ is the mass of a carbon atom (1.993 × 10^−26^ kg) [42]. The upper bound of surface density is obtained when all the vertically aligned SWNT having the same diameter are ideally close-packed. The surface density is inversely proportional to square of the diameter of SWNT and is given by Equation 5, where 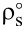 is the surface density of ideally close-packed SWNTs, *S*_SWNT_ is the area occupied by each individual SWNT, i.e., the area of a parallelogram OACB, and δ is the distance between nearest-neighbor C–C atoms in adjacent SWNTs (0.34 nm for the ideal case) [42]. The value obtained for close-packed SWNTs produced by catalytic chemical vapor deposition (CVD) is about one order of magnitude lesser than that of the theoretical value. Hence, the catalytic CVD process is nearly ideal [42].

[4]
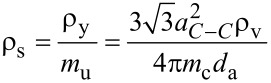


[5]
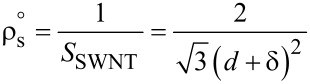


### Remedies for agglomeration

The dispersion state of nano-fillers can be tailored in two ways. Firstly, in the uncured state, the dispersion can be improved by using surfactants, mechanical mixing, or surface modification. Secondly, the dispersion state is significantly influenced by curing reactions. Details of processes that can help to avoid the agglomeration of fillers are described in the following.

#### Organic solvents

The dispersion solvent is selected for two main characteristics; (1) low viscosity and (2) the ability to lower the viscosity of polymer matrix as dispersion becomes easier in a low-viscosity medium. Due to decreased viscosity, the dispersion state of the filler can be improved. However, worsened mechanical properties were reported in some cases when an organic solvent was used in polymers [45–49]. Loos et al. used different concentrations of acetone (0, 7, 10, 13 wt %) to produce epoxy samples [50]. They observed that fracture strain, tensile strength, and Young’s modulus significantly dropped, which was attributed to residual acetone. The degradation in mechanical properties was in direct relation to the quantity of acetone used to produce epoxy samples [50]. The degraded mechanical properties may be associated with a restriction of the cross-linking process and a variation in cure kinetics due to the residual organic solvent [51]. Hong and Wu reported that the residuum of organic solvents results in lower reaction order, glass transition temperature (*T*_g_), initial curing rate, reaction rate, and a less exothermic curing reaction [52]. They further mentioned that organic solvents with lower boiling points had smaller influence on the mechanical properties and the cure kinetics of epoxy resins [52]. Therefore, the use of organic solvents has advantages and disadvantages with regard to the properties of polymer nanocomposites.

#### Dispersion methods

The nano-fillers can be dispersed in a polymer matrix using two approaches. Either external force is applied, such as sonication or mechanical stirring, to disentangle the nano-filler followed by the enclosure of dispersed nano-filler in a polymer matrix or a surfactant complex. This enclosure hinders the re-aggregation and yields a metastable dispersion. Or the nano-filler is disentangled by dispersion in a suitable solvent. In the case of graphene-based nano-fillers, the graphene sheets are separated and dissolved resulting in a polymer solution. Large quantities of CNTs can be dissolved, for instance, in superacids [36].

**Mechanical dispersion:** Various mechanical dispersion methods are summarized in Table 1 and details are provided below.

**Table 1:** Various mechanical dispersion processes.

entry	process	description	advantages	disadvantages	ref.

1	sonication (tip and bath)	The energy of high frequency sound waves is used to agitate the particles in a solution.	Equipment is inexpensive, processing is simple.	not suitable for high viscosity liquids; shortening of the filler; causes surface defects	[40]
2	calendering	A three-roll mill (3RM) uses shear forces produced in the roll gap to disperse, mix, or homogenize the viscous materials.	viscous materials can be dealt with, suitable for thermoplastic polymers	Individual nano-fillers cannot be used.	[53]
3	ball milling	The grinding action of a ball mill can unbundle the filler agglomerates.	Certain chemicals can be used for improved performance and to introduce various functional groups onto the filler.	MLG and CNT may be damaged.	[54]
4	high-shear mixing (HSM) and extrusion	HSM in general is a common dispersion technique and can be used to disperse MLG and CNT as well. Extrusion uses shear flow created by twin screws rotating at high speed.	The dispersion of fibers can be improved by high-shear mixing, and a high content of MLG and CNTs can successfully be uniformly dispersed by using this technique.	Extrusion is primarily suitable for solid materials.	[55]

**Figure 5:**
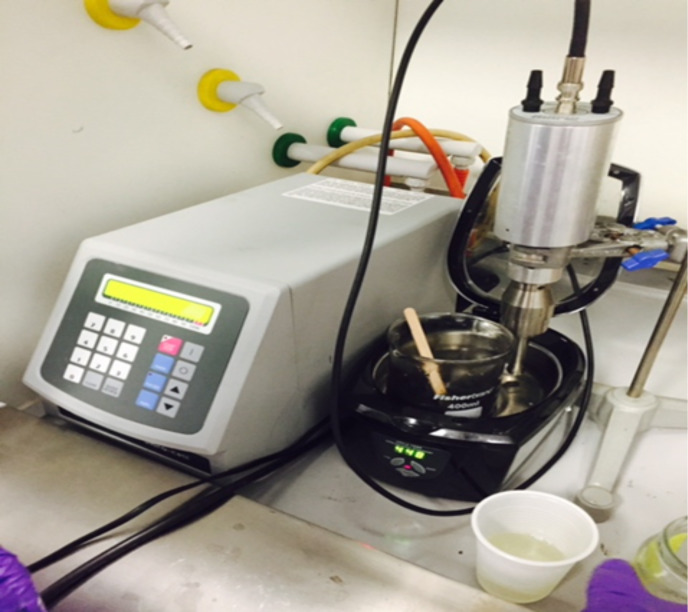
A combination of bath sonication, tip sonication, and manual stirring can help to improve the dispersion of filler in a polymer matrix.

**Sonication:** High-energy sonication may be used to uniformly disperse the MLG and CNTs in the polymer matrix. The energy of high-frequency sound waves is used to agitate the filler in a solution. It is the most common method for dispersion of nano-fillers [40]. The principle of this method is that when high-frequency sound waves are passed through a medium, attenuated waves are produced in the medium as a result. These shock waves peel off the filler one by one from the agglomerate thereby reaching uniform dispersion of the filler in a matrix. The sonication process may be aided by reducing the viscosity of the polymer using a suitable organic solvent as dispersion medium. A homogeneous composite may be obtained after solvent evaporation. Sonication is applicable for liquids with low viscosity. However, the polymers are either highly viscous or solid. So, they first need to be dissolved in a suitable solvent. There are two types of sonication devices: (1) ultrasonic bath (bath sonication), and (2) ultrasonic probe/horn (tip sonication). A combination of sonication and manual stirring can be used to disperse fillers in the polymer matrix as shown in Figure 5. Sonication may result in a shortening of CNTs and in the introduction of surface defects at sidewalls [53]. It has been reported that sonication parameters such as time and aggressiveness, if not optimized, may damage the CNTs converting them into amorphous carbon nano-fibers [40].

**Figure 6:**
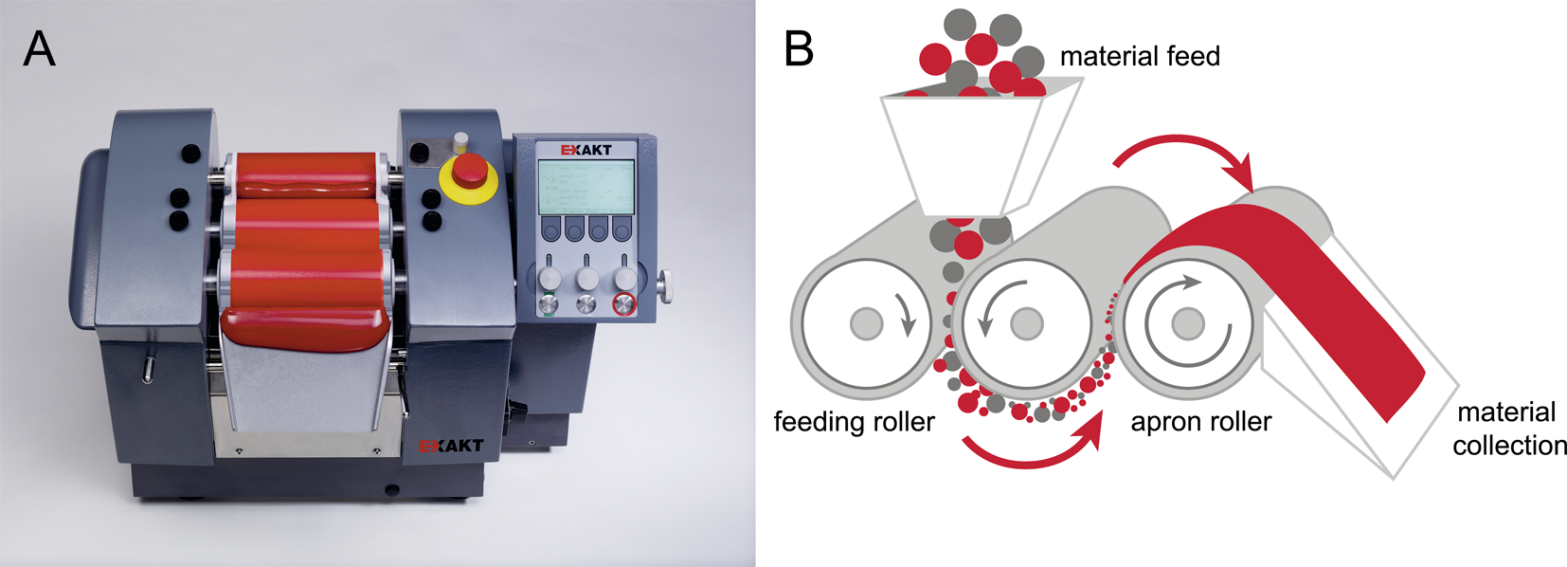
(A) Calendering mill, and (B) its working principle. Images reproduced with permission from [56], copyright 2016 EXAKT Advanced Technologies, Germany.

**Calendering:** The calender is a three-roll mill that uses the shear forces produced in the roll gaps to disperse, mix or homogenize viscous materials as shown in Figure 6. Each roll of the calender rotates at a different velocity. The first and the third roller, called feeding and apron roller, respectively, rotate in the same direction (say clockwise) while the central roller rotates in opposite direction (anti-clockwise). A knife blade removes the product from the apron roller. The process can be repeated until the desired level of mixing has been achieved [40].

The calendering process has certain limitations; the minimum gap that can be achieved between rollers is about 1–5 μm, which is nearly equal to the length of CNTs but quite large compared to the diameter of individual CNTs. So, it can only convert large agglomerates into small ones. Also, the feeding material should be present in a viscous state. It limits its applicability for thermoplastic polymers. However, it can be used to disperse the filler in liquid monomers or oligomers of thermosetting matrices, which can then be polymerized in situ to obtain nanocomposites [40].

**Ball milling:** High-quality ball mills can reduce the size of particles down to 100 nm. Ball milling can be used for the dispersion of MLG and CNT. Certain chemicals can be used for improved performance and to introduce various functional groups onto CNTs [40]. It is reported that ball milling shortens the aspect ratio of the filler [54]. Tang et al. produced highly dispersed and poorly dispersed RGO–epoxy nanocomposites using solution casting. The high dispersion of RGO in epoxy was achieved using ball milling [13]. The RGO dispersed in epoxy using sonication and not subjected to ball milling was termed as poorly dispersed. They studied the influence of graphene dispersion on the mechanical properties of the produced nanocomposite. The highly dispersed RGO–epoxy showed 52% improvement in fracture toughness (*K*_1C_) while poorly dispersed RGO–epoxy showed only 24% improvement in *K*_1C_. It shows that a better dispersion of RGO can be obtained using ball milling [13].

**Figure 7:**
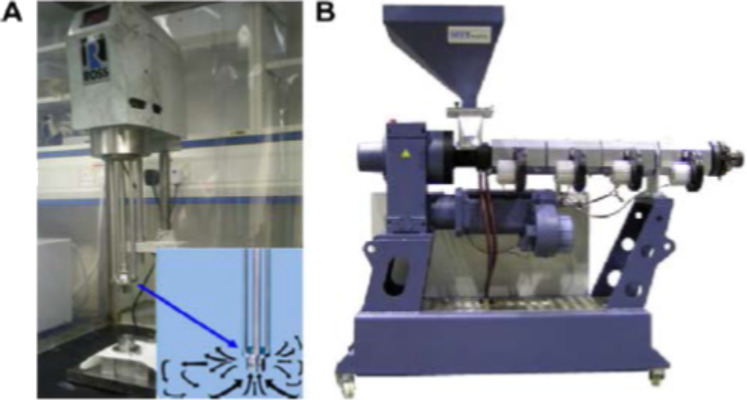
(A) Shear mixer, and (B) extruder. Reproduced with permission from [40], copyright 2010 Elsevier.

**High-shear mixing and extrusion:** High-shear mixing is a commonly used dispersion process [57]. By using high-shear mixing, the dispersion of fibers can be improved [35] and a high content of CNTs can successfully be uniformly dispersed [40]. Extrusion is another common technique for the dispersion of filler in solid polymers, such as thermoplastics, as shown in Figure 7. The thermoplastic pellets mixed with CNTs are fed through an extruder hopper. The CNT agglomerates are dispersed by shear flow created by twin screws rotating at high speed [35].

**Figure 8:**
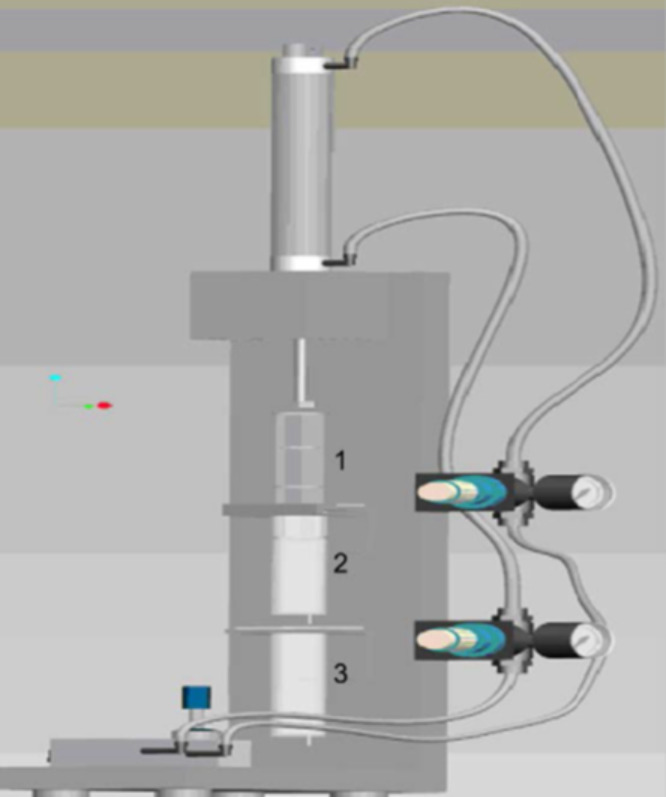
Schematic of a shearing device. Reproduced with permission from [55], copyright 2007 Elsevier.

Abdalla et al. dispersed CNTs by extrusion into EPIKOTE resin EPON 815C (bisphenol A with *n*-butyl glycidyl ether) with curing agent EPICURE 3282 containing an aliphatic amine group. The shearing device is shown in Figure 8 [55]. The mixture was extruded through syringe 2 into syringe 3 by a plunger. The process was repeated up to 50 times until a uniform dispersion was ensured [58]. The curing agent was added and the same process was repeated. The mixture was poured into a steel mold [55]. Degassing was carried out for 20 min under vacuum followed by curing for 4 h at 122 °C [58]. Meincke et al. also used a twin screw extruder to mix CVD-MWNTs, polyamide-6, and acrylonitrile butadiene styrene (ABS) at 260 °C. Pellets were made from the extrudate and test samples were made by injection molding. TEM revealed a very good dispersion of the nanotubes [59]. The nanotube powder may adhere to walls of the mixer making shear mixing difficult. A combination of melting and solution techniques could be a possible countermeasure. Tetrahydrofurane (THF) may be one option as solvent [2].

**Figure 9:**
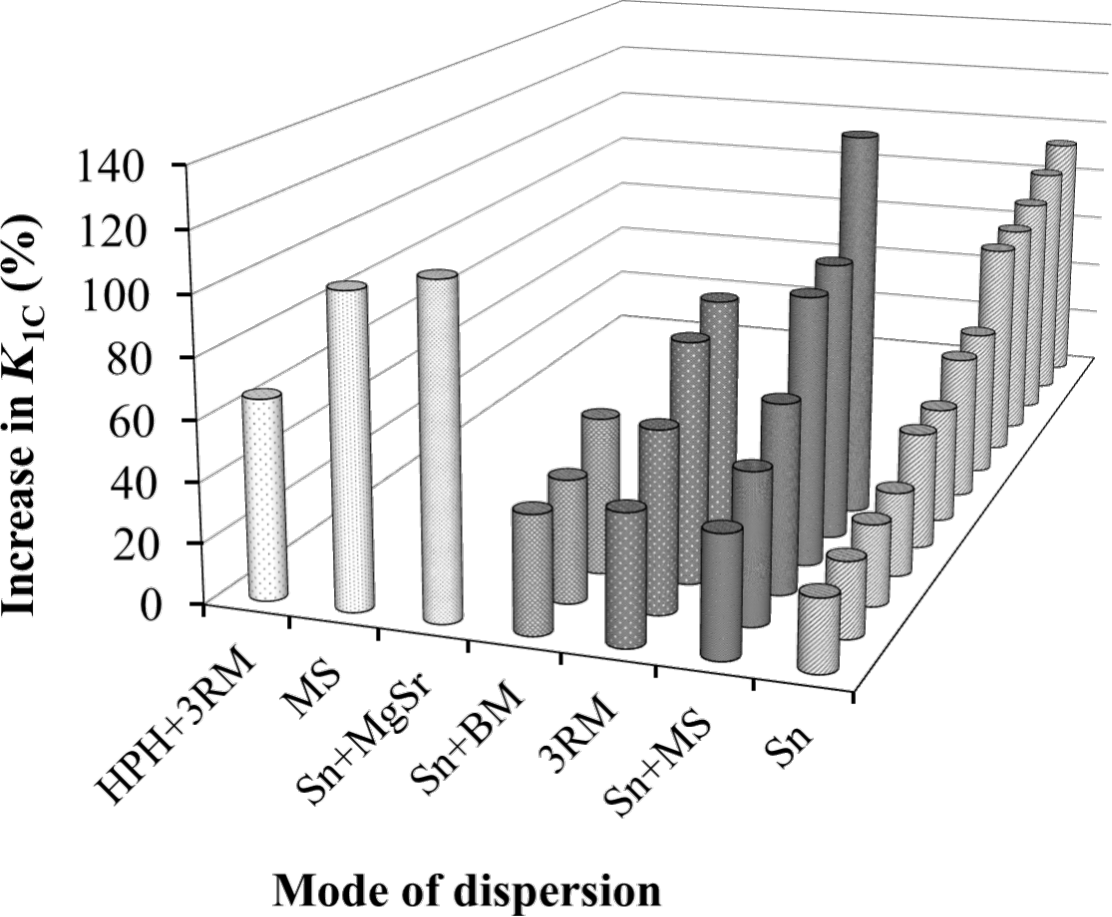
The maximum improvement in *K*_1C_ as a function of dispersion mode [17,60–62,79–104].

Several dispersion modes to disperse MLG into an epoxy matrix were successfully adopted. The maximum increase (%) of the fracture toughness *K*_1C_ as a function of dispersion mode is shown in Figure 9. In most of the cases, sonication is the main mode of dispersing reinforcements in the polymer matrix. It can be observed that when sonication is assisted by a supplementary dispersion technique such as mechanical stirring and magnetic stirring, the *K*_1C_ values were significantly increased. The maximum improvement in *K*_1C_ of 131% was achieved when a combination of sonication and mechanical stirring was employed [60]. The second highest improvement in *K*_1C_ is achieved with a combination of sonication and magnetic stirring and *K*_1C_ increased by 109% [61]. The smallest improvements of *K*_1C_ are achieved when sonication is coupled with ball milling [12,62–63]. Both sonication and ball milling reduce the sheet size and produce surface defects [64–78], and we believe that this impedes the improvement of *K*_1C_. Although calendering is an efficient way to disperse the reinforcement into the polymer matrix due to the high shear forces, the improvement in *K*_1C_ was reported to be only 86% [79], which is far below the maximum achieved with a combination of sonication and mechanical stirring.

#### Functionalization

In order to tackle the problems related to the dispersibility of MLG and CNT, surface modifications have been applied to tailor spatial distribution and/or to obtain a homogeneous dispersion in host materials [55]. Whether or not the addition MLG and CNTs improves the properties of nanocomposites is still under debate. Some studies have revealed improvements in properties through nano-fillers [105–109]. Others have observed either no improvements [110–114], or indeed a worsening of properties [45–47,115–116]. The main reason for these differences was the functionalization of MLG and CNTs that affected the dispersion and the interactions of MLG and CNTs in/with the polymer [41].

One of the key factors upon which the properties of polymer nanocomposites depend is the interfacial bond strength. The surface of graphene is very smooth, which results in weak interfacial bonding with the polymer [2]. In pristine form, MLG and CNTs are inert towards polymers and interfacial interactions are primarily based on van der Waals forces. This weak bond cannot efficiently transfer mechanical load across the filler–matrix interface. So, the surface of CNTs have been modified using two methods: (1) chemical or covalent functionalization, and (2) physical or non-covalent functionalization [40]. The different methods for the functionalization of MLG and CNTs have been summarized in Table 2.

**Table 2:** Functionalizations of CNTs and MLG.

entry	process	description	advantages	ref.

chemical or covalent functionalization				

1	organic hydrazine functionalization	reaction with organic hydrazine in an aqueous surfactant solution under argon	improved purity, solubility, and physical properties	[36,117]
2	silane functionalization	silanization in a (3-glycidoxypropyl)trimethoxysilane solution at 60–65 °C for about 6 h	Functional groups attached at the defect sites can undergo further chemical reactions	[53]
3	strong acids	treatment of MLG and CNTs with a mixture of sulfuric acid and nitric acid causes functionalization of MLG and sidewalls of CNTs	Oxygenated side groups exert electrostatic repulsive forces causing exfoliation.	[40]
4	oxidation	surface oxidization by heat treatment in oxygen or air, plasma treatment, chemical treatment, and ozone treatment	decrease of contact angle	[55,58]

physical or non-covalent functionalization				

5	surfactant functionalization	Surfactants are physically absorbed on the surface of the filler.	lowers surface tension of filler, avoids filler segregation	[40]
6	endothermal	atoms or molecules are inserted inside the CNTs by capillary action through defect sites	can significantly improve thermal and electrical conductivities	[40]

**Figure 10:**
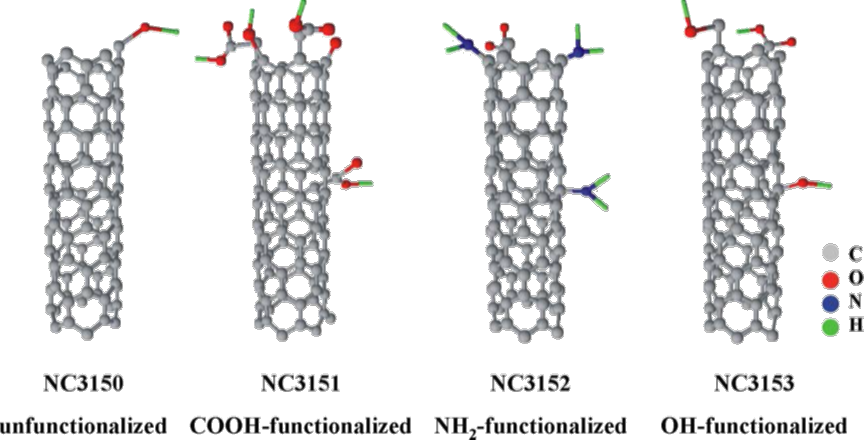
Unfunctionalized and differently functionalized CNTs. Reproduced with permission from [53], copyright 2010 Elsevier.

**Chemical or covalent functionalization:** Chemical functionalization of CNTs is the attachment of chemical groups either at the ends or at the sidewalls [55,57]. The different reactions for functionalization include cycloadditions such as the Diels–Alder reaction and the addition of azomethine ylides, carbene and nitrene addition, chlorination, bromination and hydrogenation [40]. Different chemically attached functional groups on the sidewalls of nanotubes are shown in Figure 10 [53]. Nayak et al. [118] carried out the solvent-free side-wall functionalization of SWNTs with 4-vinylaniline through atom transfer radical polymerization. Different functional groups yield varying interfacial interaction strengths with the polymer matrix [118].

MLG and CNTs can be grafted covalently into polymers using two main strategies: (i) “grafting from” and (ii) “grafting to” [2]. In the “grafting from” approach, initiators are initially immobilized onto the surface of the filler. The fillers are bound with desired polymer molecules by in situ polymerization. The main advantage of this method is that a high grafting density can be achieved. However, this method is very sensitive to the processing parameters [2]. Using the “grafting to” method, polymer molecules and fillers are functionalized with suitable functional groups, which are then chemically reacted to form a bond. The main advantage is that processing is relatively easy. However, the grafting density is low due to slow diffusion. Also, this approach can be applied for polymers with reactive functional groups [2].

The SWNTs can be functionalized by reacting them with organic hydrazine in an aqueous surfactant solution. The reactions involved are carried out in argon atmosphere [117]. A typical disorder band in Raman spectrum gives proof of the sidewall functionalization. This sidewall functionalization not only improves purity and solubility, but it also changes the physical properties. In fact, the introduction of heteroatoms in SWNTs can lead to novel properties. Heating may remove the attached functionalized groups. The functionalized SWNTs can be dissolved in organic solvents up to about 100 mg/L. The functionalization of SWNTs in liquid ammonia by reductive alkylation using lithium and alkyl halides can make the SWNTs soluble in common organic solvents [36]. Functionalization with diazonium compounds can make SWNTs water-soluble [36]. The differently functionalized CNTs show varying dispersibility in different surfactants [2,54,57]. However, it has been shown that functionalization does not necessarily result in an increased dispersibility. Amino-functionalized CNTs are difficult to disperse compared to non-functionalized CNTs [53].

Another covalent functionalization technique is defect functionalization. At the defect sites of MLG and CNTs, functional groups such as –COOH (carboxylic acid) and –OH (hydroxyl) are attached. Defects can be any structural deviations, such as pentagons and heptagons in the hexagonal graphene structure, and oxygenated sites. Defects may also be produced by reaction with strong acids such as HNO_3_, H_2_SO_4_ or their mixture, strong oxidants such as KMnO_4_, ozone and reactive plasma. The functional groups attached at the defect sites of MLG and CNTs can undergo further chemical reactions including but not limited to silanation, thiolation, esterification, polymer grafting, alkylation and arylation. Functionalization changes the nature of the CNTs from hydrophobic to hydrophilic and it can strengthen the CNT–polymer bond [40].

The functionalization agent for silane functionalization of MLG and CNTs is (3-glycidoxypropyl)trimethoxysilane (GPTMS) [41]. The silanization is carried out in a GPTMS solution at about 60–65 °C for about 6 h [41]. The silane-grafted CNTs showed a marked improvement of dispersion in the polymer matrix [41]. The silane-treated CNTs showed greater improvements in flexural modulus and strength, fracture resistance, and thermal stability than untreated CNTs [41]. There are improved interfacial interactions between the silane-functionalized CNTs and the epoxy matrix due to strong covalent bonding [41].

**Figure 11:**
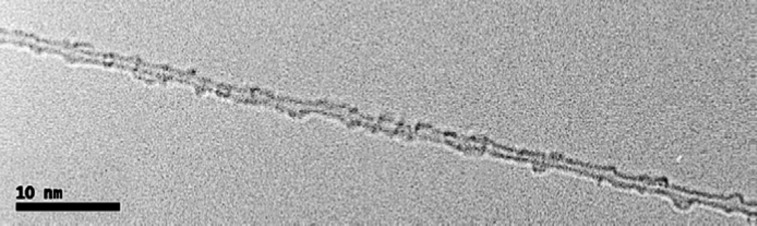
TEM image of a SWNT; the uneven surface shows the attachment of functionalized groups. Reproduced with permission from [36], copyright 2009 Elsevier.

The treatment with sulfuric acid and nitric acid also causes functionalization of MLG and CNTs. These oxygenated side groups exert electrostatic repulsive forces causing exfoliation. However, this acid treatment results in a shortening of the filler. The plasma-treated CNT exhibit superior properties compared to acid- and amine-treated nanotubes [55]. Figure 11 shows a TEM image of a chemically functionalized SWNT. The uneven surface shows the functional groups [36]. The chemical functionalization causes sp^3^ hybridization and damages the CNTs causing a shortening and producing surface defects which deleteriously affect the electrical properties and ordering of SWNTs in films and fibers [36,57]. Also, chemical functionalization can reduce the maximum nanotube buckling force by up to about 15% thereby deteriorating the mechanical properties of nanocomposites [2].

**Oxidation of SWNTs:** The oxidation of CNTs surface is possible by, for instance, heat treatment in oxygen and air, plasma treatment, chemical treatment and ozone treatment. Oxidation can also be carried out in lithium aluminum hydride solution [41]. Table 3 shows different oxidation methods for CNTs taken from Abdalla et al. [55,57], Chen et al. [119] and Siddiqui et al. [120].

**Table 3:** Oxidation steps for CNTs.

step number	reference		
	[55,57]	[119]	[120]

1	treating with sulfuric/nitric acid mixture (3:1) to remove impurities	treatment with nitric acid to remove amorphous carbon and to insert carboxyl groups (100 mg MWNT in 20 mL concentrated nitric acid)	bath sonication of CNT in acetone for 20 min
2	sonication in water bath for 3 h at 40 °C	heating for 2 h under reflux	filtration
3	dilution with distilled water (1:5 by volume)	washing with water	drying in a vacuum oven
4	filtration through polycarbonate membrane filter (0.8 µm pore size)	multiple filtrations through poly(tetrafluoroethylene) membranes	oxidation in a UV/O_3_ chamber for 1 h
5	washing with water	dispersion in water and freeze drying	further functionalization with triethylenetetramine (TETA)
6	drying in a vacuum oven for 24 h		bath sonication of CNT with TETA in excess amount (60 °C, 30 min)
7	results in –COOH groups attached to the CNT surfaces		filtration
8			removal of untreated amine by washing with acetone

The conventional oxidation process for CNTs was developed by Liu et al. [121]. SWNTs (1 g) are dispersed in 250 mL of 3:1 H_2_SO_4_ (98%)/HNO_3_ (70%) mixture followed by sonication for about 1 h and stirring at room temperature for about 3 h for moderate oxidation and to maintain a high aspect ratio. After washing with deionized water, HCl is added to produce carboxylic acid groups. Again, the mixture is washed with deionized water, while the pH value is maintained at 5–6. Then the aqueous suspension is centrifuged and carboxy-functionalized CNTs (SWNT–COOH) are obtained. These are then dried overnight in a vacuum oven at about 90 °C [122]. To oxidize and create active halves on the surface of CNTs through ozone treatment, CNTs are exposed to ultraviolet (UV) light in an ozone chamber [41]. Due to the ozone treatment, the concentration of oxygen on the CNT surface increases, which decreases the contact angle. The lower contact angle results in an improvement of interfacial interactions and leads to an enhancement of mechanical properties such as tensile strength, modulus and coefficient of friction [123]. Eitan et al. attached carboxy groups on CNTs and then dispersed these surface-modified CNTs in an epoxy resin without curing agent. Spectroscopic and thermal analysis showed covalent interfacial bonds [124].

**Physical or non-covalent functionalization:** Supermolecular complexes of filler are formed through wrapping the filler with polymers. The wrapping process involves π–π interactions and van der Waals interactions [2,40]. Surfactants have also been used to functionalize MLG and CNTs. Surfactants are physically adsorbed on the surface of CNTs. It lowers the surface tension of MLG and CNTs diminishing the driving force for the formation of aggregates. The CNT dispersion can be enhanced by non-ionic surfactants in case of water-soluble polymers [40]. Both ionic and non-ionic aqueous surfactant solutions can be used to disperse the CNT in low concentrations. Examples of surfactants include sodium dodecylbenzene sulfonate (SDBS), sodium dodecyl sulfate (SDS), and sodium deoxycholate [36].

**Figure 12:**
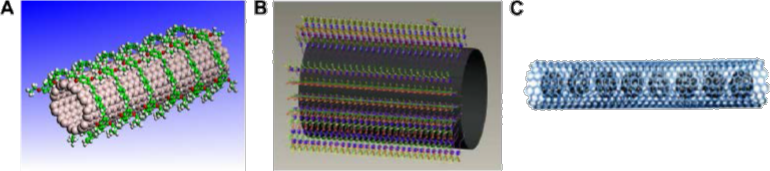
Schematic of non-covalent CNT functionalizations: (A) polymer wrapping, reproduced with permission from [40], copyright 2010 Elsevier; (B) surfactant absorption, reproduced with permission from [40], copyright 2010 Elsevier; and (C) endohedral CNT, reproduced with permission from [126], copyright 2002 John Wiley and Sons.

The most commonly used surfactants are derivatives of SDS [2]. Yan et al. modified SWNTs with surfactants Volan and BYK-9076 to improve the dispersion state in the polymer matrix [125]. They incorporated the treated SWNTs as secondary reinforcement in glass fiber reinforced epoxy and reported an increase in flexural strength of up to 16%, which can be attributed to the improved dispersion state, absence of agglomerates, and strong interfacial interactions. The glass transition temperature did not change [125]. The synthesis of endohedral CNTs (Figure 12c) is a physical modification in which the foreign atoms or molecules are inserted inside of the CNT by capillary action through defect sites [40].

**Figure 13:**
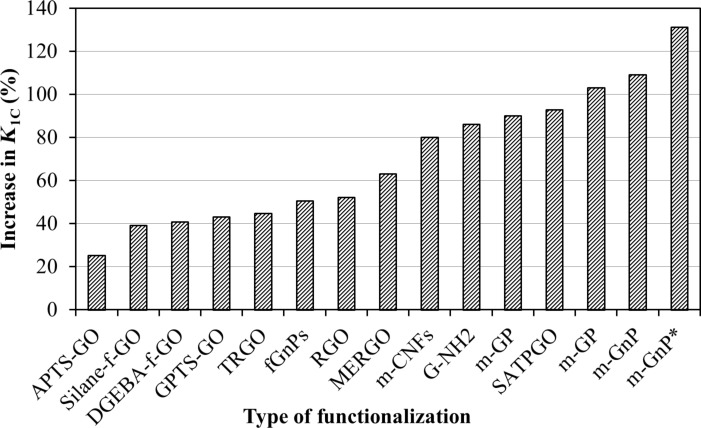
Improvement in *K*_1C_ as a function of functionalization method [17,60–62,79–104].

The influence of the different functionalization methods on *K*_1C_ values is shown in Figure 13. The smallest improvement was achieved for amino-functionalized graphene oxide (APTS-GO) [90], while the largest improvement was recorded for surfactant-modified graphene nanoplatelets [60].

**SWNTs in superacids:** Strong acids such as fuming sulfuric acid and clorosulfonic acid can dissolve and disperse MLG and CNTs in large quantities provided that the fillers have not been surface stabilized [36]. The superacids cause protonation of the filler surface, which produces electrostatic repulsion resulting in filler dispersion [36]. The protonation can be measured from the shift in Raman peaks once the filler is dispersed in the superacids [36]. The protonation is completely reversible by adding water [36], and HCl is used to control the pH value of the dispersion [2]. The key difference between treating CNTs with superacids and surfactants is that the superacids dissolve CNTs, while surfactants stabilize the CNT dispersion. SWNTs can be dissolved in HSO_3_Cl within minutes [36].

#### Synthesis

There are three different methods for the production of CNTs: (1) arc discharge, (2) CVD, and (3) laser ablation. The size, shape, yield, structure and orientation of CNTs and MLG are largely dependent on the process variables. Therefore, fine-tuning of the variables is required to obtain fillers with desired features. Some of these process variables are discussed in the following.

**Arc discharge:** MWNTs were first observed in the arc discharge reaction of a fullerene reactor. This method was later employed to produce SWNTs [2,36]. Arc discharge can be used to produce MWNTs with very few defects [2] and the production of large quantities is possible at low cost [127]. However, they contain a large amount of impurities such as graphite fragments, amorphous carbon, polyhedral carbon and metal catalyst particles. The carbonaceous impurities are removed from the arc-discharge soot usually by refluxing in HNO_3_ or thermal annealing in oxygen-containing atmosphere. The metal catalyst particles are removed by treatment with inorganic acids [127].

**Figure 14:**
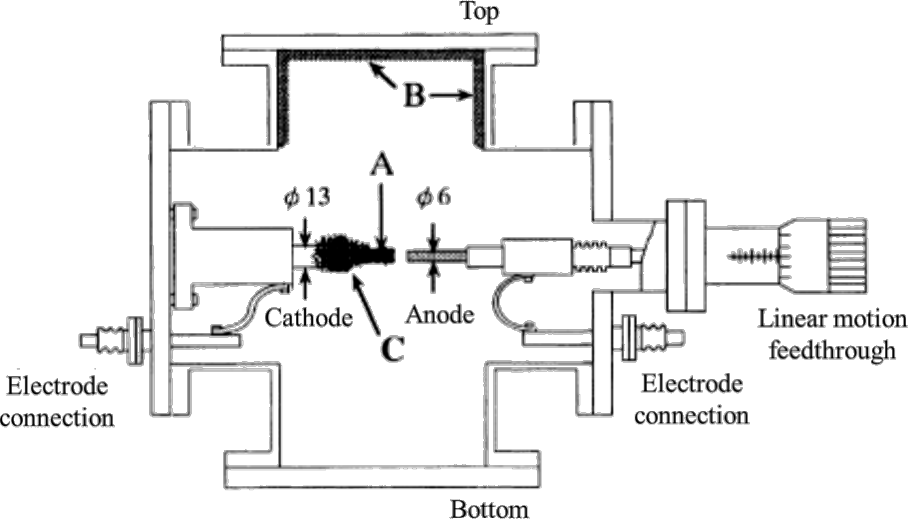
Schematic diagram of an arc evaporator (horizontal arrangement of electrodes). (A) carbonaceous hard deposit grown on the end of the cathode called “cylindrical hard deposit”, (B) soot grown on surface of the cathode is “chamber soot”, (C) soot deposited on the ceiling of the evaporator is “cathode soot”. Reproduced with permission from [128], copyright 2000 Elsevier.

Figure 14 shows the arc discharge apparatus used by Saito and Uemura for the production of CNTs. The electrodes are graphite rods (99.99% pure) to produce MWNTs, while a carbon anode containing metal particles is used to produce SWNTs. The anode and cathode are 50 mm long each with diameters of 6 mm and 10–13 mm, respectively. The discharge current and voltage were fixed, respectively, at 70 A and 20 V. The surface temperatures of anode and cathode were ca. 4000 K and ca. 3500 K, respectively. Because of this temperature difference, the anode gets corroded while the cathode remains intact. A translation feedthrough was used to position the anode tip to maintain the optimum electrode spacing (ca. 1 mm) [128].

About 50% of the carbon vapors condense at the cathode tip as slag-like deposit (arrow A in Figure 14) called “cylindrical hard deposit”. Some of the remaining carbon vapors condense in the gas phase and form soot. It adheres to the reaction chamber walls (arrow B in Figure 14) and is called as “chamber soot”. The remaining carbon vapors condense at the tail of “cylindrical hard deposit” and are called “cathode soot” (arrow C in Figure 14). CNT can be grown on fibers by CVD method [129]. The CNT-grafted fibers have inferior tensile properties. It is mainly because of the surface defects introduced during grafting the CNT on fibers through CVD. The deleterious effect of CNT grafting depends upon the nature of fiber, the surface treatments and growth conditions [129]].

**Chemical vapor deposition:** The most commonly used synthesis of CNTs is the reaction of a gaseous carbon feedstock on catalyst particles, i.e., chemical vapor deposition (CVD) [2,36]. CNT can be produced by using organo-metallic compounds as precursor (e.g., ferrocene), a carbon feedstock (e.g., toluene) and a carrier gas (e.g., hydrogen) [130]. It is difficult to control the diameter of the nanotubes. However, CVD is an economical method, favorable for mass production, and easy to scale up for commercial production [2]. Other processes are fluidized bed CVD, catalytic gas flow CVD, and the growth of CNT carpets from embedded catalyst particles in a substrate. One of the most common techniques is the HiPco (high pressure CO) process. It is a CVD technique that does not use catalyst particles for CNT growth. It is a relatively economical and easily scalable process [2,36].

Most of the aforementioned methods yield CNTs of shorter lengths ranging between 0.05 and 3 μm [36]. CNTs can be prepared by spray pyrolysis process in which ferrocene acts as precursor and hexane is used as carbon source [131]. Spray pyrolysis is very simple and does not involve harmful ingredients such as benzene. Hexane is used in the process which is a good solvent for ferrocene. Hexane suppresses the formation of impurities and results in a greater yield of pure CNTs making it quite suitable for commercial scale production.

Certain CVD techniques yield control over the number of walls of MWNTs and over the defect density [36]. The catalytic gas flow technique has a higher CNT yield than substrate-growth techniques [36]. The methods using catalysts supported on a substrate are non-continuous, which makes them unsuitable for industrial scale-up [131]. The point-arc microwave plasma chemical vapor deposition (PAMP-CVD) technique has been used to produce densely-packed and vertically aligned (DPVA) SWNTs with the currently highest volume and surface densities of 60–70 kg·m^−3^ and 10^16^ m^−2^, respectively [44].

**Laser ablation:** Laser ablation was employed to produce fullerene. It was later applied to produce SWNTs on metal particles as catalyst. The high price of CNTs limits their widespread application. This is mainly caused by limited mass production [130]. Laser ablation is capable of the production of SWNTs in large quantities with average diameters of about 1.2 nm [36]. Laser ablation produces refined CNT but at a lower yield.

#### Composites

Some of the processes to produce nanocomposites are described in Table 4. The properties of composite systems have been significantly improved, but such processes are hardly feasible to scale up due to cost, time, and equipment considerations. Vice versa, processes that can be scaled up easily, only marginally improve the composite properties. To increase the volume fraction of MLG and CNTs or to improve the degree of alignment and dispersion complicates the manufacturing process making it less probable to scale up industrially [132]. In 2014, 2009 research papers were published with “graphene” and “epoxy” in their title (Thomson Reuters). Out of 2009 articles, about 830 articles were on graphene–epoxy nanocomposites produced using solution casting. Therefore, solution casting technique is still the preferred route for the production of polymer nanocomposites [133].

**Table 4:** Various production routes for polymer nanocomposites.

entry	process	description	advantages	disadvantages	ref.

1	solution mixing	Filler is first dispersed in a solvent and then in a polymer followed by casting or precipitation.	equipment is economical; processing is simple; most common method	solvent traces are detrimental; inapplicable to insoluble polymers	[40]
2	melt blending	Filler is dispersed in a polymer at high temperature by applying high shear forces.	does not involve any solvent; suitable for thermoplastic polymers	suitable only for low filler content	[53]
3	in situ polymerization	Filler is mixed with monomers, which are then subjected to addition or condensation polymerization.	results in covalent bonding improving interfacial interactions; grafting of polymer macromolecule is possible on nanotube walls; suitable for high filler loading, for unstable and insoluble polymers, and for any MLG/CNT–polymer combination	requires reaction chamber for polymerization reactions, outgassing is required	[40]
4	latex technology	Filler can be dispersed in polymers that are either produced by emulsion polymerization or can be brought in the form of emulsion. The filler is added after polymerization and not in the monomers.	easy, viscous polymers can be used; water is used as solvent making the process cost effective and environmently friendly	only applicable to emulsions/latex	[40]
5	solid freeform fabrication (SFF)	SFF covers a family of manufacturing processes in which components are manufactured layer by layer.	alignment of CNT is possible		[35]
6	extrusion freeform fabrication (EFF)	Materials are subjected to extrusion to manufacture a component.	fiber alignment is possible; tensile test specimens can be made; significant improvement in properties; in situ polymerization possible		[35]

**Other methods:** Fused deposition modeling is an extrusion-based technique and can be used to improve the alignment of fibers in a polymer matrix [35]. Some other methods include wet lay-up method [132], injection molding, electrospinning, coagulation, spinning of coagulant, densification, layer-by-layer deposition and evaporation [2,40].

#### Filler alignment

The mechanical properties of CNT–polymer composites are strongly influenced by the alignment of the CNTs in the matrix [134]. An increase of the modulus of the composite up to a factor of five for perfectly aligned fibers has been observed [2]. The maximum enhancement of mechanical properties can be achieved by aligning the reinforcement, i.e., making the property changes anisotropic [135]. The enhancement is then maximum along the orientation axis of the filler and minimum in the transverse direction [2]. There are two factors that govern the degree of CNT alignment in the polymer matrix: (1) the diameter of CNTs, and (2) the CNT content. Smaller diameters and lower content improves the CNT alignment [40].

The fiber alignment can be improved by fiber spinning. It yields anisotropic composite properties, but an improvement of the mechanical properties of the CNT–polymer nanocomposites was shown [35]. Haggenmuller et al. used fiber spinning to align SWNTs in a poly(methyl methacrylate) (PMMA) matrix [136]. They observed an increase in yield strength and draw ratio of the composite fiber. The yield strength was doubled and the draw ratio was increased from 40 to 300 in 5 wt % SWNT–PMMA system [35]. Kumar et al. also used fiber spinning to align SWNTs in poly(phenylene benzobisoxazole (PBO) matrix and found an improvement of mechanical properties [137]. Along with an increase of tensile strength, SWNTs can increase the strain prior to failure. Nanofiber alignment is suitable to improve strength and modulus. However, it was observed that results were not in accordance with the rule of mixtures. The factors to cause this deviation include fiber alignment, interfacial bonding, and morphology [35].

Methods for the alignment of CNTs include DC plasma-assisted hot filament CVD, melt processing, mechanical stretching and shearing, electroplating, and application of electric and magnetic fields [134]. In one study, it was shown that making a thin film by extrusion and drawing it before the heat gets dissipated helps to achieve the alignment of CNTs. The inter-tube slippage can be avoided through nano-mechanical interlocking by growing the CNTs in the form of coils using reduced-pressure catalytic CVD [138]. Vertically aligned MWNTs are commonly grown using catalytic thin films. These catalytic films can easily be produced by conventional physical techniques such as sputtering and evaporation [44]. To increase the degree of CNT alignment, the manufacturing of nanocomposites should be carried out at the lowest possible temperature. Elevated temperatures lower the viscosity, which results in CNT scattering. CNTs can be aligned magnetically because of their anisotropic magnetic susceptibility [134]. Magnetic fields may be applied during the curing process, which makes it impossible to completely align the whole CNT fraction in the polymer matrix.

#### Hybrid nano-fillers

Addition of hybrid nano-fillers not only improves the dispersion states of MLG and CNTs in the polymer matrix. In addition, synergistic effects become active that help to improve the physical properties of hybrid nanocomposites. Sumfleth et al. doped titania into MWNT–epoxy [57]. They found enhanced CNT dispersion and synergistic effects in these multiphase nanocomposites. Ma et al. doped nanoclay into CNT–acrylonitrile butadiene styrene (ABS) system and found enhanced CNT dispersion [139]. Nanoclay also improved the CNT dispersion state in CNT–polyamide nanocomposites. Titania can improve the mechanical properties of polymers. So, titania is a better option to improve the dispersion of CNTs than block copolymers. Also, large amounts of CNTs can be uniformly dispersed using titania. The addition of nanoparticles in nanocomposites can also improve their thermal stability (*T*_g_), which is an important requirement for structural applications [57].

### Properties of composites

Out of 2009 research articles about graphene published in 2014, 830 articles were related to the synthesis of epoxy–graphene nanocomposites. In addition, almost in all cases, the nanocomposites were produced using solution casting. Therefore, solution casting and thermosetting epoxy are still the favorite synthesis methods. The first report about the preparation of aligned CNT–polymer composites was published in 1994 by Ajayan et al. [140]. Tomohiro et al. modified carbon-fiber reinforced epoxy composite with length-controlled cup-stacked CNTs and determined the mechanical properties [141]. Auad et al. produced SWNT–epoxy elastomers and showed that the nanocomposites had superior damping capacity in an extended temperature range [122]. The addition of CNTs as secondary reinforcement in glass fiber-based polymer composites can significantly improve the resistance against cyclic delamination and crack propagation. The inter-laminar fracture toughness also improves due to the deflection of the cracks by CNTs. The fatigue life can be increased up to three times when in-plane cyclic loading is applied. CNTs can decelerate the crack propagation and delamination as energy is dissipated to pull-out and break the CNTs. In addition, CNTs cause crack bridging, which helps improve the mechanical properties [132]. The *z*-axis properties of laminated nanocomposites can also be improved by CNTs through direct reinforcement of the polymer matrix, toughening effect and fiber bridging [142]. The influence of MLG and CNTs on the mechanical, thermal, electrical, and damping properties is discussed in the following sections.

#### Mechanical properties

The mechanical properties of polymer nanocomposites depend strongly on filler dispersion state, aspect ratio, alignment, and on the interfacial bonds. In addition, topography and morphology of the filler also influence the mechanical properties of nanocomposites. Atif et al. showed that a wide particle size distribution yields an effective reinforcement as the empty spaces created by the larger particles can be occupied by the smaller particles thereby resulting in a strong network of the filler and a concomitant increase in the mechanical properties [143]. A uniform dispersion of the filler in the matrix is the most important issue. Aggregated filler acts as stress concentrator and severely deteriorates the mechanical properties. Nano-fillers can be assembled in the matrix in different ways. It has been shown that nano-sized hydroxyapatite (HA) particles are organized in the form of “lines” in tooth enamel while a uniform dispersion was observed in bone mimic [1]. There is a certain critical value of the filler content below which the composite properties are improved. Above, the properties are, in some cases, even inferior to those of the matrix alone mainly because of the poor dispersion state [40]. The nanocomposite strength can be used to estimate the dispersion state of the filler [120].

The interfacial interactions should be such that efficient transfer of mechanical load is guaranteed [2]. The aspect ratio should be large for a better load transfer from matrix to reinforcement. The impact strength and fracture toughness increase significantly while elastic modulus and tensile strength increase marginally with increase in aspect ratio [40]. The transfer of external loads also requires strong interfacial bond. Qian et al. have studied the load transfer properties using TEM in polystyrene–1 wt % MWNT. They have reported a 42% increase in breaking strength, which is an indication of strong interfacial adhesion [144].

**Figure 15:**
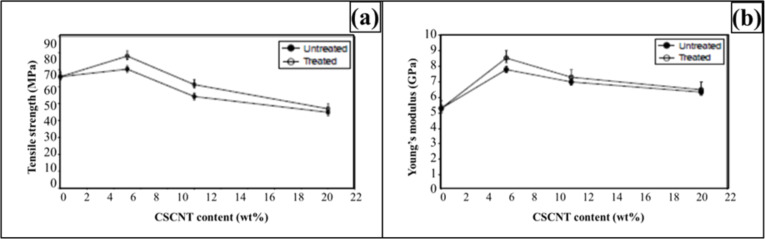
Effect of CSCNT loading on strength and modulus of nanocomposites. Reproduced with permission from [141], copyright 2009 Elsevier.

The trend for strength and modulus with varying content of CSCNT loading is shown in Figure 15 [141]. It can be seen that mechanical properties improved with increasing CSCNT loading until a critical value is reached after which the effect is reversed. It is mainly because of the formation of aggregates and voids. Similarly to aggregates, the voids act as stress concentrators and deteriorate the mechanical properties.

**Figure 16:**
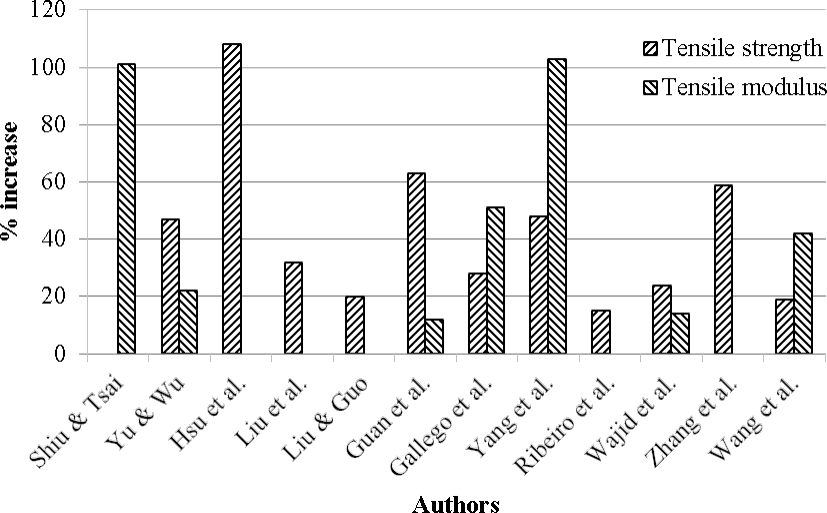
Increase in tensile properties of epoxy–graphene nanocomposites [145–146,149–158].

MLG can significantly improve the mechanical properties of epoxy nanocomposites. The percent improvements in tensile strength and tensile modulus are shown in Figure 16. The maximum improvement in tensile strength is as high as 108% [145] and tensile modulus up to 103% [146]. MLG was also found to improve the flexural properties of nanocomposites. Naebe et al. produced covalently functionalized MLG–epoxy nanocomposites and reported 18% and 23% increase in flexural strength and modulus, respectively [147]. Qi et al. produced graphene oxide–epoxy nanocomposites and reported increase up to 53% in flexural strength [148].

The impact strength and hardness were also significantly improved by graphene in epoxy nanocomposites. For example, Ren et al. applied a combination of bath sonication, mechanical mixing, and shear mixing to disperse GO in cyanate ester–epoxy and produced nanocomposites using in situ polymerization [159]. They reported an increase of 31% in impact strength. Qi et al. produced GO–epoxy nanocomposites and reported increase in impact strength up to 96% [160], whereas Lu et al. produced GO–epoxy nanocomposites and reported increase in impact strength up to 100% [161]. Shen et al. produced graphene nanosheet–epoxy nanocomposites and reported an increase in impact strength up to 11% [162] and Bao et al. reported increase in hardness up to 35% [163]. The critical energy strain rate (*G*_1C_) also improved with the incorporation of graphene in epoxy nanocomposites. Meng et al. produced epoxy–graphene nanocomposites and reported increase in *G*_1C_ up to 597% [164].

#### Electrical properties

In MLG- and CNT-reinforced polymer nanocomposite, there is change in electrical behavior from insulator to conductor at a certain critical filler content known as percolation threshold [40,54]. The percolation theory was mainly established for particulate reinforcement; so, it has limitations for fiber reinforcement. The percolation theory was modified and extended for fiber fillers to incorporate the effect of aspect ratio by many researchers including Balberg and collaborators [165–167], Bug et al. [168], Munson-McGee [169], Philipse [170], Celzard et al. [171] and Neda et al. [172]. The CNTs provide a continuous path for electrons to flow. The percolation threshold depends upon the following factors: (1) dispersion state, (2) filler aspect ratio, (3) processing route, (4) curing conditions, (5) temperature, (6) structural quality of filler, (7) distribution of individual filler, and (8) external electrical/magnetic fields [40,54,57].

MLG and CNTs can be used to make polymers conductive at a very low percolation threshold [2]. In epoxy nanocomposites, the percolation threshold can be as low as 0.1 wt % of CNT [57]. The lowest percolation threshold value reported to date is 0.005 wt % MWNTs [54]. It is worth mentioning that fibers have a much lower percolation threshold value than particles [54]. A better filler dispersion lowers the percolation threshold [120]. The CNT dispersion can be estimated from the electrical conductivity values of nanocomposites [57]. A decrease in electrical conductivity indicates a better filler dispersion [57]. To cut short the cost of polymer nanocomposites, low-cost fillers are also added. MLG and CNTs form a scaffold and other conducting fillers facilitate the flow of electrons along the scaffold [40].

The lowest percolation threshold is achieved for non-functionalized CNTs [54]. Functionalization lowers the aspect ratio of CNTs. It also improves interfacial bonding that wraps a thin matrix layer around the CNTs and acts as insulating barrier. Any process reducing the aspect ratio, such as functionalization and sonication, results in an increased percolation threshold [54]. The density of nanotubes increases with increasing diameter. SWNTs have lower density than MWNTs. If volume fraction is taken into account, the lowest values for the percolation threshold can be achieved with nanoparticles having the highest densities [54]. MWNTs have higher densities than SWNTs and are preferable to raise the electrical conductivity of nanocomposites. MWNTs also have a lower surface area than SWNTs, which yields a better dispersion and, in turn, a higher electrical conductivity [54].

Liu and Grunlan suggested a synergistic percolation behavior after the addition of nanoclay in SWNT–epoxy resulting in a reduced percolation threshold [57]. The incorporation of silica in carbon black–epoxy, and graphite nanoplatelets in CNT–epoxy resulted in improved electrical conductivity values [57]. The electrical conductivity decreases with the addition of titania in MWNT–epoxy. This decrease in electrical conductivity becomes significant when the amount of CNTs is less than that of titania. It can be explained on the basis of zeta-potential. When titania reacts with carboxylic acids and anhydride hardener during the curing process, it gets negatively charged. On the other hand, in organic solvent, MWNTs have a positive zeta-potential. There is electrostatic attraction between oppositely charges particles. The attachment of titania at the MWNT surface reduces the van der Waals forces among nanotubes because of the exposed free surface resulting in an improved dispersion state [57].

**Figure 17:**
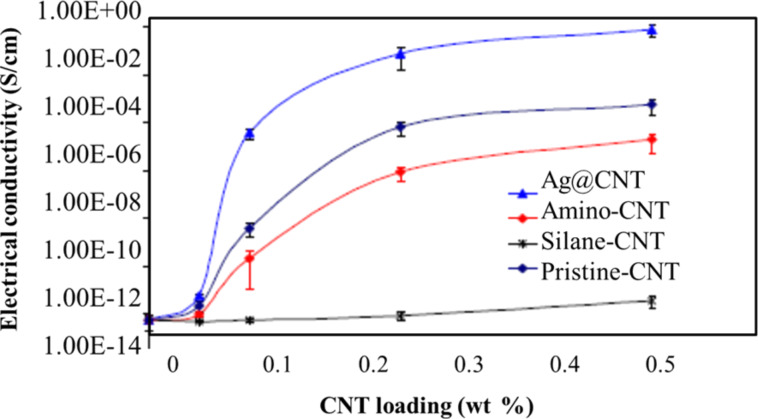
Effect of CNT functionalization on the electrical conductivity of CNT–epoxy nanocomposites [40], copyright 2010 Elsevier.

The electrical conductivity increased by nine orders of magnitude by adding untreated CNT up to 0.50 wt % [41]. However, in case of silane–CNT, the conductivity increased only by two orders of magnitude. This is in agreement with previous works as shown in Figure 17 [40]. There are two main reasons for this observation. Firstly, there is wrapping of the CNTs by polymer through strong covalent bonding which perturbs the π-electrons of CNTs. This effect is more pronounced for well-dispersed CNTs. Secondly, the probability of formation of electrical networks decreases as the dispersion state of CNT improves due to functionalization [41]. Also, different functional groups affect the electrical conductivity in different ways.

**Figure 18:**
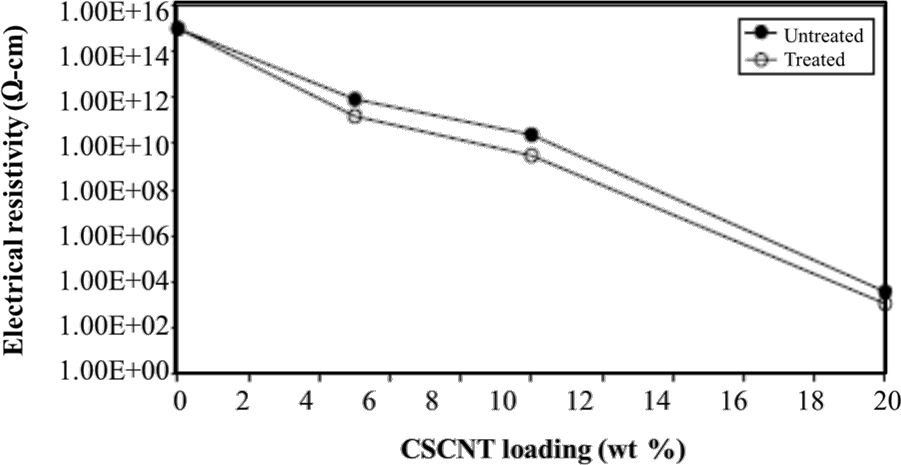
Effect of CSCNT loading on electrical resistivity. Reproduced with permission from [141], copyright 2009 Elsevier.

The effect of CSCNT loading on the electrical resistivity is shown in Figure 18 [141]. The electrical resistivity decreased with increasing CSCNT loading. It can be noted that ozone treatment did hardly influence the electrical properties of the nanocomposites.

**Figure 19:**
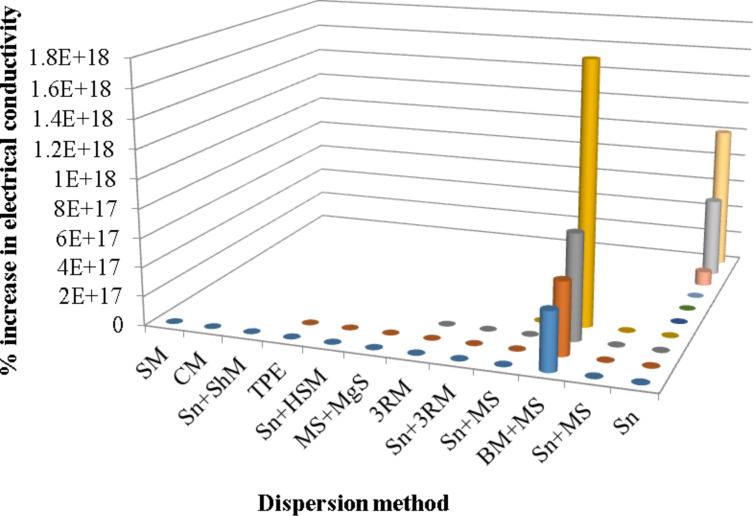
Increase in electrical conductivity as a function of dispersion method [70,97,173–201].

The electrical properties reported for MLG–epoxy nanocomposites were studied as a function of the dispersion mode (Figure 19). The maximum improvement in electrical conductivity was observed in case of a combination of ball milling and mechanical stirring.

#### Thermal properties

The high thermal conduction of graphene can be utilized to manufacture thermally conductive polymer nanocomposites [2]. However, the thermal properties of MLG/CNT–polymer nanocomposites were only marginally improved in many reported cases. This is because phonons prefer to travel through the matrix and not along the CNTs [40]. However, Gojny et al. suggest that phonons will preferably travel through CNTs in CNT–epoxy nanocomposites. The crystalline graphite lattice provides a long free path to phonons, while there are only few phonon vibrational modes in amorphous epoxy. It makes CNTs a preferred route for phonon conduction [54].

The heat conduction through polymer nanocomposites depends greatly upon the interface resistance. The strong interfacial bonds inhibit phonon transport [40]. A strong interfacial bonding increases the coupling losses and damps the phonon amplitude resulting in a reduced increase in thermal conductivity of nanocomposites. So, weak interfacial bonding is preferable for a significant increase in thermal conductivity of nanocomposites [54]. However, this is under debate [202]. A covalent bond is formed between fluorinated MWNTs and the epoxy matrix, which enables phonon transport and improves the thermal conductivity [134]. The interface thickness marginally influences the thermal conductivity of nanocomposites [202]. There is linear relationship between the transverse thermal conductivity of CNTs and the nanotube length. It has been shown that the influence of the nanotube length on the thermal conductivity of nanocomposite is small for short lengths, while it is significant for long nanotubes. Knibbs has shown that the properties in the longitudinal direction of the nanocomposite are determined by the fibers while the properties in transverse direction are dictated by the matrix [134].

The thermal stability of PVC–MWNT was found to improve with increasing MWNT content. It has been shown that the increase in thermal conductivity is highest when the orientation of CNTs is along the direction of heat flow [134]. The factors affecting the phonon conduction through CNTs include boundary surface scattering phonon active modes, inelastic Umkla scattering, and free path length [54].

The properties of semi-crystalline polymers highly depend upon crystallization behavior. CNTs improve the crystallization rate of polyether ether ketone (PEEK) because they act as heterogeneous nucleation sites [203]. The curing enthalpy of the nanocomposite increases with increasing concentration of CNTs. The curing degree is given by Equation 6. CNTs can exhibit the phenomenon of electromagnetic shielding thereby reducing curing degree with increasing CNT content [204].

[6]
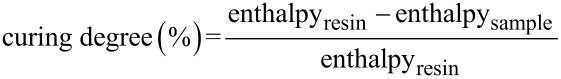


Warrier et al. introduced CNTs in a glass fiber–epoxy system in three ways [205]. They added CNTs in the epoxy matrix, in the sizing formulation of the glass fibers, and finally combined the former two. They studied thermo-physical properties (co-efficient of thermal expansion, CTE, and glass transition temperature, *T*_g_) and interlaminar fracture toughness (mode I). The CTE was reduced in longitudinal direction up to 31% by sizing glass fibers with CNTs. However, it increased along the transverse direction. The CTE of the CNT-containing epoxy matrix system was higher than that of the pure epoxy system mainly because of the accumulation of CNT at the fiber–matrix interface resulting in decrease in obstacles for expanding polymer chains. The dimensions of the CNTs are similar to polymer chains, which affects the alignment of polymer chains thereby increasing the polymer *T*_g_. The glass fibers sized with CNT in pure epoxy showed the maximum rise in *T*_g_, which was 11%. The nanocomposite containing CNT in the epoxy matrix showed a rise in *T*_g_ of 8–9%.

The highest thermal conductivity of nanocomposites can be achieved with un-functionalized CNTs with the lowest interfacial area, i.e., MWNTs. SWNTs have a large surface area. It causes large interfacial boundary scattering of electrons and phonons resulting in low enhancement of the conductivity values. The inner layers of MWNTs are not in contact with the matrix. Thus, coupling losses are minimized and the conductivity values are significantly increased. The phonon scattering during transfer from CNT to CNT also reduces the overall CNT impact on thermal conductivity of nanocomposites. MWNTs with the largest aspect ratio and diameter are most suitable to improve the thermal conductivity of nanocomposite because of low surface area, low coupling to the matrix, and low surface scattering at the CNT–polymer interface [54].

**Figure 20:**
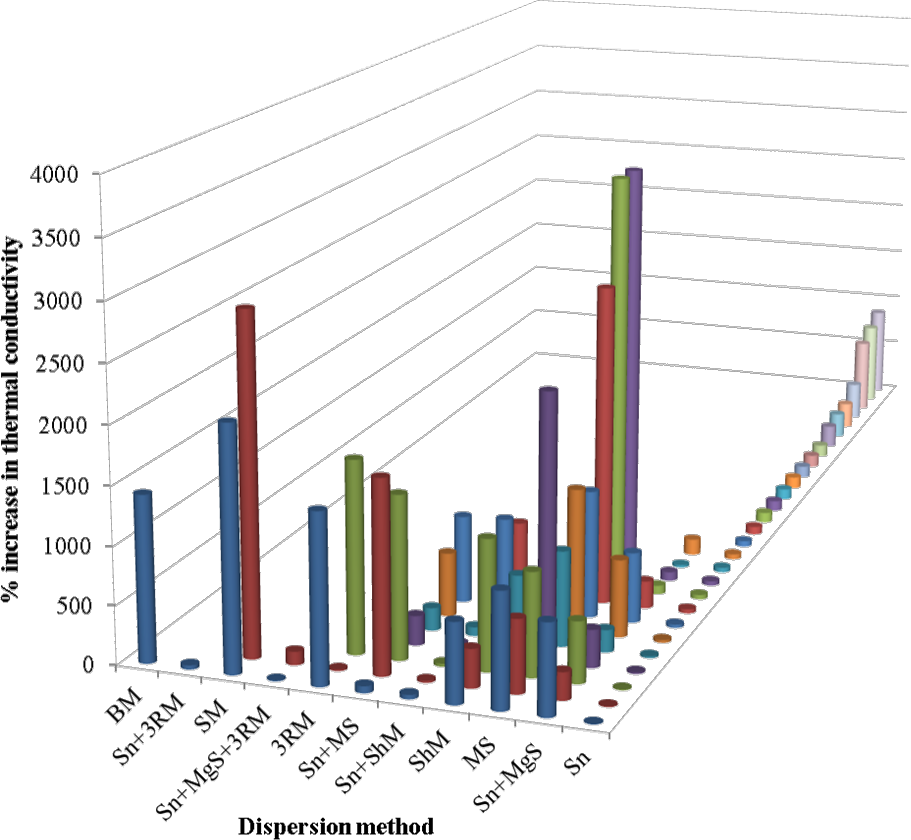
Increase in thermal conductivity as a function of the dispersion method [45,89,111,195,198,206–220].

The thermal properties reported for MLG–epoxy nanocomposites were studied as a function of dispersion mode (Figure 20). The highest improvement in thermal conductivity was observed after mechanical stirring. In general, sonication caused a lower improvement in thermal conductivity. However, maximum improvement in thermal conductivity (not shown in Figure 20) was observed after sonication and is (1.6 × 10^4^)% [199]. It can be observed that both thermal and electrical conductivities improved after mechanical stirring.

#### Damping properties

Noise mitigation and vibration damping in machines and structures is very essential and requires the employment of special materials with high damping capacity. Along with high damping capacity, a material should be lightweight and have a high stiffness, particularly for aerospace industry applications. It has been shown that the damping capacity of stiff epoxies (as well as epoxy elastomers) can be enhanced by the addition of CNTs [122]. A weak interfacial bonding enhances the damping ability of CNT–polymer nanocomposites. It can be explained by the “stick–slip” theory. When the CNT–polymer nanocomposite is subjected to an external load, shear stresses are generated at the CNT–polymer interface because of the difference in the elastic properties of CNT and polymer. Initially, CNT and matrix deform equally. But, after certain critical value of load depending upon interfacial bond strength, debonding takes place and the matrix deforms more than the CNTs. The matrix flows over the CNT surface. The frictional forces between CNTs and matrix dissipate the deformation energy increasing the damping properties. SWNTs are more effective in increasing the damping properties than MWNTs. It is because the inner layers of MWNTs do not offer friction to dissipate energy. There can be an increase in the damping ratio of up to 1400% by the addition of 50 vol % CNTs in an epoxy matrix, with reference to neat epoxy [135]. The maximum improvement in damping properties can be achieved by uniform dispersion of the filler [40].

## Conclusion

Based on the critical analysis the following conclusions can be established:

1. Most of the synthesis methods for MLG and CNTs produce them in entangled form. Due to high aspect ratio, the disentanglement becomes difficult. The disentanglement further becomes difficult due to the presence of van der Waals forces. Therefore, the dispersion of MLG and CNTs is an arduous task. This task turns into a challenge when the objective is to disperse them in viscous polymers. Therefore, a meticulous tuning of processing parameters is essential to avoid aggregation and achieve uniform dispersion.
2. There are various factors that influence the dispersion state of MLG and CNTs including, but not limited to, surface chemistry, synthesis methods, diameter, shape, size and type of polymer matrix used.
3. To achieve a uniform dispersion state of MLG and CNTs in the polymer matrix, organic solvents are commonly used such as acetone, ethanol and DMF. The dispersion of MLG and CNT is much easier in low-viscosity organic solvents than in viscous polymers. However, a complete removal of organic solvent through evaporation is essential. Any residuum of organic solvent will cause porosity, which degrades the performance of nanocomposites.
4. The volume fraction of the MLG and CNTs also influences their dispersion state. Due to very high surface area of CNT (1300 m^2^/g) and MLG (2500 m^2^/g), it is very difficult to achieve uniform dispersion beyond a certain volume fraction. Due to the high number of factors controlling the dispersion state, it is not easy to delineate one specific value of volume fraction above which the dispersion state starts getting poor. Most of the literature reported that mechanical properties improve up to 1 wt % of MLG and CNTs and start degrading when the filler content is increased above 1 wt %.
5. The other factor defining the dispersion state is the dispersion method employed. The most common dispersion methods include calendering [89], sonication [221], and mechanical stirring [85].
6. The most important and widely studied method to improve dispersion state is the functionalization. It is because it not only improves the dispersion state, but also improves the interfacial interactions.
7. Another way of improving the dispersion state is the use of hybrid nano-fillers. The improvement in dispersion state is related to the synergistic effects between nano-fillers such as titania and CNTs [57].
